# Ring-Augmented versus Non-Ring-Augmented One-Anastomosis Gastric Bypass as Revisional Surgery after Sleeve Gastrectomy: a Comparative Cohort Study

**DOI:** 10.1007/s11695-026-08616-2

**Published:** 2026-04-13

**Authors:** Ahmed Elmasry, Mohamed Sallam, Mahmoud F. Abdelaziz, Amr Abdelatyy, Mohamed Abdelgawad, Muhammad S. Sahali, Eslam E. Mohamed, Hashem Altabbaa, Ahmed El Shamarka, Mohamed H. Zidan

**Affiliations:** 1 Arab Contractors Medical Centre, Cairo, Egypt; 2 Independent Surgeon, Ministry of Health and Population, Cairo, Egypt; 3 Matareya Teaching Hospital, Cairo, Egypt; 4 Al-Basheer-Hospital, Amman, Jordan; 5 The Research Papyrus Lab, Alexandria, Egypt; 6 Alexandria University, Alexandria, Egypt

**Keywords:** Ring-augmented one-anastomosis gastric bypass, Revisional bariatric surgery, Sleeve gastrectomy failure, Recurrent weight gain, Banded OAGB

## Abstract

**Background:**

Sleeve gastrectomy (SG) is the most frequently performed metabolic and bariatric surgery (MBS) worldwide, but long-term suboptimal weight loss (SOWL) or recurrent weight gain (RWG) affects up to one third of patients and often necessitates revisional procedures. One-anastomosis gastric bypass (OAGB) is an effective revision, yet pouch dilation and late RWG remain concerns. Ring augmentation (RA) has been proposed to enhance restriction and sustain weight loss, but evidence in the revisional OAGB setting is limited.

**Methods:**

This retrospective comparative cohort study analyzed 94 adults who underwent revisional OAGB after SG at a high-volume tertiary MBS center. Patients were grouped into two categories: Ring Augmented OAGB (RaOAGB) (*n* = 45) and Non-Ring Augmented OAGB (NRa-OAGB) (*n* = 49). The primary outcomes were percentage excess weight loss (%EWL), percentage total weight loss (%TWL), and change in body mass index (BMI) at 3, 6, and 12 months. Secondary outcomes included improvement of obesity-related conditions, metabolic parameters (lipid profile, HbA1c), nutritional markers, operative time, hospital stay, and early/late complications.

**Results:**

Both groups achieved significant weight reduction at 12 months (*p* < 0.001 within groups). RaOAGB yielded greater BMI reduction (27.6 vs. 32.2 kg/m²), %EWL (81.7% vs. 61.3%), and %TWL (31.5% vs. 24.2%) compared with NRa-OAGB (all *p* < 0.001). Lipid profiles improved in both groups; LDL decreased, and total cholesterol equalized by 12 months. Glycemic control changes were modest and similar between cohorts. Operative time, length of stay, and early postoperative complications were comparable. Ring-related adverse events were infrequent (food intolerance 6.7% vs. 4.1%; no erosions were observed; one patient (2.2%) required ring removal at 10 months due to persistent vomiting and food intolerance).

**Conclusions:**

RaOAGB is a safe and effective revisional option for RWG and SOWL after SG, achieving significantly greater 1-year weight loss than standard OAGB, with uncommon ring-related adverse events, including one ring removal. These findings support the role of mechanical reinforcement to enhance the durability of revisional bypass but warrant confirmation through long-term, multicenter randomized trials.

**Supplementary Information:**

The online version contains supplementary material available at 10.1007/s11695-026-08616-2.

## Key Points

Ring-augmented one anastomosis gastric bypass (raOAGB) achieved greater 12-month weight loss than non-augmented OAGB (NRa-OAGB), with rare ring-related adverse events.

Both procedures produced comparable metabolic improvements and safety profiles.

Operative time and hospital stay were similar between groups.

Ring-related adverse events were rare; one patient required ring removal.

raOAGB is a safe and effective revisional option after failed sleeve gastrectomy, meriting long-term multicenter validation.

**Supplementary Information:**

The online version contains supplementary material available at 10.1007/s11695-026-08616-2.

## Introduction

Obesity is a chronic disease that affects over 800 million adults worldwide and is associated with significant morbidity, mortality, and healthcare costs [1]. Among the available interventions, sleeve gastrectomy (SG) has become the most commonly performed primary metabolic and bariatric surgery (MBS) globally, known for its effective early weight loss and metabolic enhancements alongside relative technical simplicity [2, 3].

However, the challenge of long-term weight recurrence remains significant [4–7]. Literature indicates that 10% to 49% of SG patients experience suboptimal weight loss (SOWL) or recurrent weight gain (RWG) within five to seven years after SG, with around 20% to 30% necessitating revisional surgery [4–7]. Several anatomical and physiological factors, including sleeve dilation, neo-fundus development, gastro-gastric fistula formation, and hormonal adaptations, likely contribute to these suboptimal outcomes [8, 9].

In patients with RWG or SOWL after SG, revisional surgery is increasingly indicated. One-anastomosis gastric bypass (OAGB) has gained traction as a preferred option due to its operative efficiency and consistent weight loss outcomes, along with favorable metabolic effects [10, 11]. A recent head-to-head meta-analysis and a network meta-analysis revealed that OAGB performed after SG achieves comparable or superior excess weight loss and resolution of obesity-related conditions compared to Roux-en-Y gastric bypass (RYGB), while maintaining the streamlined single-anastomosis approach [10, 11]. Nonetheless, concerns persist regarding long-term pouch dilation and late weight recurrence, paralleling issues seen in primary bypass surgeries like RYGB [12–17].

To address these risks, ring augmentation (RA) has been proposed. This technique involves the placement of a fixed, non-adjustable silicone ring around the gastric pouch, aimed at preserving mechanical restriction and preventing dilation over time [12, 13, 16, 18–20]. Long-term outcomes from ring-augmented RYGB (raRYGB) [21] suggest enhanced weight loss durability, while preliminary data on ring-augmented OAGB (raOAGB) [22] indicate possible improvements in initial weight reduction [21, 22]. However, these advantages must be weighed against potential increases in ring-related complications, and the evidence in the revisional context remains limited.

The ongoing RiMini trial and similar studies are expected to yield comparative data in primary settings; however, their relevance to revisional cases remains uncertain [23]. This study seeks to evaluate the outcomes of raOAGB versus non-augmented OAGB (NRa-OAGB) in the context of revisional MBS following SG. By investigating weight-loss durability, metabolic outcomes, and complication profiles within a real-world cohort, we aim to ascertain whether the theoretical benefits of RA translate into measurable clinical outcomes in this increasingly prevalent scenario.

## Methods

### Study Design and Setting

This is a retrospective, comparative cohort study conducted at Arab Contractor Hospital, Cairo, Egypt, a high-volume tertiary MBS center. The study aimed to compare clinical outcomes between raOAGB and NRa-OAGB, following primary SG. Eligible cases were identified from a prospectively maintained institutional MBS database. The study period spanned from January 2021 to January 2024, and reporting adheres to the Strengthening the Reporting of Observational Studies in Epidemiology (STROBE) guidelines [24].

### Participants

This study included adult patients (≥ 18 years) who had previously undergone primary SG but required a revisional OAGB due to RWG or SOWL. RWG was defined according to the IFSO consensus as a regain of > 30% of the maximum weight loss achieved after SG, relative to nadir weight. SOWL was defined as < 20% total weight loss, in accordance with IFSO reporting standards [25]. Eligibility criteria dictated a baseline body mass index (BMI) exceeding 30 kg/m² with at least one obesity-related disease, or a BMI greater than 35 kg/m², irrespective of obesity-related disease, adhering to the ASMBS/IFSO 2022 guidelines [26]. Additionally, a minimum follow-up duration of 12 months from the index SG was required. All revision indications were confirmed through multidisciplinary team assessment after failure of structured non-surgical measures.

Exclusion criteria included any prior MBS procedure other than SG, such as adjustable gastric banding, RYGB, or vertical banded gastroplasty. Patients were also excluded if preoperative esophagogastroduodenoscopy (EGD) revealed Barrett’s esophagus, severe esophagitis, or diffuse gastritis. Additional exclusions included active pregnancy at the time of revision, or any psychiatric or behavioral condition likely to impair adherence to postoperative follow-up.

Patients were stratified into two cohorts according to the revisional technique, with the raOAGB group undergoing placement of a non-adjustable MiniMizer Gastric Ring^®^ (Bariatric Solutions International, Switzerland), and the NRa-OAGB group undergoing the procedure without ring augmentation.

### Surgical Techniques

All revisional procedures were performed laparoscopically by a single experienced MBS team following standardized institutional protocols. Pneumoperitoneum was established via optical trocar entry, with careful dissection due to adhesions from prior SG. Standard port placement included three 12-mm ports (camera, right, and left working channels) and two 5-mm ports (liver retraction and assistant).

Adhesiolysis around the gastric sleeve was carried out using a bipolar vessel-sealing device (LigaSure™, Medtronic, USA). In our standardized conversion protocol, resizing of the gastric pouch is performed routinely in all patients. A long, narrow, vertical pouch was then fashioned along the lesser curvature from the level of the incisura up to the angle of His and calibrated over a 38 French bougie.

Gastrojejunostomy was performed in a side-to-side, stapled fashion using a 30 mm linear stapler, placed 150–200 cm distal to the ligament of Treitz. The enterotomy was closed using a 3 − 0 barbed absorbable suture (V-Loc™, Medtronic, USA) in a continuous running fashion, starting at the biliary limb and aligned with the pouch staple line.

In the raOAGB group, a MiniMizer Gastric Ring^®^ (7.5 cm circumference; Bariatric Solutions International, Switzerland) was loosely positioned 3–5 cm distal to the gastroesophageal junction and secured directly onto the staple line using non-absorbable sutures through integrated fixation sites [18]. The passage route (pars flaccida versus perigastric) was selected according to individual perigastric adiposity; however, the target ring level (3–5 cm distal to the gastroesophageal junction), looseness, and fixation method were standardized in all cases. Perigastric fat was minimized by targeted clearance at the intended ring level using blunt and bipolar dissection to expose the gastric wall/staple line and ensure direct ring–gastric wall contact before fixation, to reduce the likelihood of postoperative loosening or slippage during weight loss. Particular care was taken to avoid excessive ring tension, consistent with expert recommendations to minimize the risk of dysphagia, ischemia, and ring-related adverse events, including erosion [18]. The introducer was removed once the ring was properly positioned [18]. A video demonstrating our technique can be found at Video 1.

To prevent complications such as dysphagia, ischemia, or food intolerance, ring tension was intentionally kept loose, maintaining mechanical restriction without compromising gastric compliance [18].

Staple line reinforcement was performed in all patients using absorbable 3 − 0 polydioxanone sutures (PDS^®^, Ethicon, Johnson & Johnson, USA). Posterior cruroplasty, when indicated for hiatal hernia, was performed using non-absorbable 2 − 0 braided polyester sutures (Ethibond^®^ Excel, Ethicon, Johnson & Johnson, USA). Concomitant cholecystectomy was performed as needed using the same port configuration. Intraoperative air leak testing was routinely conducted via orogastric insufflation.

Postoperative care, including pharmacologic thromboprophylaxis, early ambulation, proton pump inhibitor therapy, structured dietary progression, and multivitamin supplementation, was standardized across both groups.

### Data Collection and Variables

Data were retrospectively extracted from a prospectively maintained institutional MBS registry. Collected variables included baseline demographics (age, sex), anthropometrics (weight and BMI at the time of SG and at revision), and the interval in months between the primary and revisional procedures. Obesity-related conditions such as type 2 diabetes mellitus (T2DM), hypertension (HTN), dyslipidemia, and obstructive sleep apnea (OSA) were documented based on preoperative clinical assessments. Dyslipidemia was treated as a physician-documented baseline comorbidity recorded in the registry and was not re-classified using baseline lipid panel thresholds.

Operative data included revisional technique (raOAGB vs. NRa-OAGB), operative time, identification of hiatal hernia, and performance of concomitant procedures such as posterior cruroplasty or cholecystectomy. Intraoperative adverse events, if present, were recorded.

Primary outcomes included weight loss expressed as percentage of total weight loss (%TWL), percentage of excess weight loss (%EWL), and BMI change at 3, 6, and 12 months following revisional surgery. Secondary outcomes comprised changes in obesity-associated diseases and postoperative nutritional markers, including hemoglobin, ferritin, albumin, calcium, vitamin B12, and vitamin D levels, measured preoperatively and at 12 months postoperatively.

Patient-reported outcomes were evaluated utilizing the Suter et al. Food Tolerance Questionnaire, a validated tool designed to assess tolerance across various food consistencies, the frequency of emesis, and overall eating satisfaction [27]. The questionnaire was administered in person during routine postoperative follow-up visits at 6 and 12 months, aligning with laboratory assessments and nutritional counseling sessions. Scores were computed following the original methodology outlined by Suter et al., resulting in a composite food tolerance score ranging from 0 to 27, which facilitated between-group comparisons.

Postoperative complications were classified as early, within 30 days, or late, beyond 30 days. Ring-related adverse events, including vomiting, dysphagia, or the need for ring removal, were specifically evaluated in the raOAGB cohort.

### Power Analysis

A priori power analysis was conducted using Jamovi statistical software (The jamovi project, version 2.3, 2022) to determine whether the study sample size was sufficient to detect a clinically meaningful difference between groups. With group sample sizes of 45 and 49 participants, respectively, and assuming a two-sided significance level of α = 0.05, the study design achieves a statistical power of 0.844 to detect an effect size of δ ≥ 0.62. This corresponds to an 84.4% probability of correctly rejecting the null hypothesis when a true effect of at least this magnitude exists, indicating that the sample size provides adequate sensitivity to detect moderate group differences.

### Statistical Analysis

All statistical analyses were conducted using IBM SPSS Statistics for Windows, Version 25.0 (IBM Corp., Armonk, NY, USA). The Shapiro–Wilk test was used to assess the normality of distribution for continuous variables. Variables with normal distribution were expressed as mean ± standard deviation (SD) and compared between groups using the independent samples t-test. Non-normally distributed variables were reported as median with interquartile range (IQR) and analyzed using the Mann–Whitney U test.

Categorical variables were summarized as frequencies and percentages, with between-group comparisons performed using Pearson’s chi-square test or Fisher’s exact test, as appropriate.

Intra-group comparisons over time (baseline vs. 12 months) for continuous variables were conducted using the Wilcoxon signed-rank test. A two-tailed p-value < 0.05 was considered statistically significant. Two distinct p-values were reported: P1 for between-group comparisons (raOAGB vs. NRA-OAGB) at each follow-up point, and P2 for within-group comparisons from baseline to 12 months.

## Results

### Baseline Characteristics

A total of 94 patients underwent revisional one-anastomosis gastric bypass (OAGB), of whom 45 underwent raOAGB, and 49 underwent the non-augmented variant. The two groups were similar in terms of mean age (39.51 ± 10.62 years for raOAGB vs. 40.59 ± 10.03 years for NRa-OAGB, *p* = 0.613) and sex distribution (22 (48.9%) females in raOAGB and 31 (63.3%) in NRa-OAGB, *p* = 0.212) (Table 1). However, the NRa-OAGB group had a significantly higher proportion of ASA III patients (30 (61.2%) vs. 17 (37.8%), *p* = 0.012), indicating a greater preoperative anesthetic risk. Hypertension was significantly more prevalent in the NRa-OAGB group compared to the raOAGB group (31 (67.4%) vs. 15 (33.3%), *p* = 0.004), and the same applied to GERD (18 (36.7%) vs. 6 (13.3%), *p* = 0.010) (Table 1). In contrast, dyslipidemia was significantly more common among raOAGB patients (23 (51.1%) vs. 6 (13%), *p* < 0.001) (Table 1). Baseline lipid profile values are reported separately as objective laboratory measurements (Table 2) and may not directly mirror comorbidity documentation in a retrospective registry. No significant differences were observed between groups for type 2 diabetes, asthma, sleep apnea, and shortness of breath (Table 1).

**Table 1:** Baseline demographic and preoperative clinical characteristics of patients undergoing ring-augmented one-anastomosis gastric bypass (raOAGB) versus non-ring-augmented one-anastomosis gastric bypass (NRa-OAGB) as revisional procedures after sleeve gastrectomy

		raOAGB	NRa-OAGB	Test statistic	*P*-value
		(*n* = 45)	(*n* = 49)		
Age, year^*^		39.51 ± 10.62	40.59 ± 10.03	t= −0.507	0.613
Sex					
Female		22 (48.9)	31 (63.3)	FE(𝒳^2^ = 1.971)	0.212
Male		23 (51.1)	18 (36.7)		
ASA Score					
ASA I		5 (11.1)	0 (0)	𝒳^2^ = 8.822	**0.012**
ASA II		23 (51.1)	19 (38.8)		
ASA III		17 (37.8)	30 (61.2)		
Type 2 Diabetes Mellitus		13 (28.9)	12 (24.5)	FE(𝒳^2^ = 0.233)	0.648
Hypertension		15 (33.3)	31 (67.4)	FE(𝒳^2^ = 8.410)	**0.004**
Asthma		7 (15.6)	12 (24.5)	FE(𝒳^2^ = 1.161)	0.315
Shortness of breath		18 (40)	15 (30.6)	FE(𝒳^2^ = 0.907)	0.391
Obstructive sleep apnea		9 (20)	14 (28.6)	FE(𝒳^2^ = 0.933)	0.350
Dyslipidemia		23 (51.1)	6 (13)	FE(𝒳^2^ = 16.61)	**< 0.001**
GERD		6 (13.3)	18 (36.7)	FE(𝒳^2^ = 6.756)	**0.010**

^*^ mean ± SD. Values are presented as n (%) unless otherwise indicated. Age is expressed as mean ± standard deviation (SD). P-values refer to comparisons between raOAGB and NRa-OAGB, using an independent-samples t-test for age, Pearson’s chi-square test for categorical variables with sufficient cell counts, and Fisher’s exact test (FE) when expected counts were < 5

*ASA* American Society of Anesthesiologists, *GERD* gastroesophageal reflux disease, *T2DM* type 2 diabetes mellitus

**Table 2:** Laboratory parameters at baseline, 6 months, and 12 months after revisional ring-augmented OAGB (raOAGB) and non-ring-augmented OAGB (NRa-OAGB), including hematologic, glycemic, lipid, and nutritional indices

		Pre-operative (Baseline)	6 months	12 months
Hemoglobin (g/dL)	raOAGB	12.7 (12.3–13.85.3.85)	12.4 (11.65–12.5)	12.2 (11.46–12.9)
	NRa-OAGB	13.2 (12.65–13.85)	12.3 (11.6–12.5)	12.2 (11.3–12.8)
	P1-value	0.157	0.587	0.646
Glycated Hemoglobin (HbA1c) %	raOAGB	5.4 (4.8–6.8)	-	5.1 (4.75–5.8)
	NRa-OAGB	5 (4.9–6.45)	-	5.2 (5.1–5.9)
	P1-value	0.946	-	0.057
Triglycerides (mg/dL)	raOAGB	151 (140.3–165)	142.5 (133–159)	136 (133–139.8)
	NRa-OAGB	195 (168–234.5)	-	150 (130–170)
	P1-value	**< 0.001**	-	**0.004**
Total Cholesterol (mg/dL)	raOAGB	186 (167–198)	176 (157–189)	166 (150.5–180)
	NRa-OAGB	222 (198–242)	182 (168–190)	167 (153–175)
	P1-value	**< 0.001**	**0.037**	0.931
High-Density Lipoprotein (HDL) (mg/dL)	raOAGB	53.1 (50.3–55.8)	54.6 (53.3–60.8)	62 (56.6–64.6)
	NRa-OAGB	40 (30.5–63)	-	101 (91–120)
	P1-value	0.072	-	**< 0.001**
Low-Density Lipoprotein (LDL) (mg/dL)	raOAGB	122.5 (101–128)	110 (96.3–122)	89 (86–98)
	NRa-OAGB	140 (132.5–160)	-	89 (77–101)
	P1-value	**< 0.001**	-	0.473
Vitamin B12 (pg/mL)	raOAGB	310 (270–375)	380 (320–425)	360 (280–400)
	NRa-OAGB	320 (265–390)	380 (325–440)	360 (295–415)
	P1-value	0.590	0.570	0.275
Serum Calcium (mg/dL)	raOAGB	8.9 (8.5–9.6)	8.9 (8.5–9.12)	8.69 (8.46–9.76)
	NRa-OAGB	9.2 (9–9.4.4)	9 (8.8–9.2)	8.9 (8.7–9.15)
	P1-value	**0.028**	0.055	0.512

Values are presented as median (Q1–Q3). P1 denotes between-group comparisons (raOAGB vs. NRa-OAGB) at each time point using the Mann–Whitney U test. Where 6-month values are not reported, only baseline and 12-month data were available

*HbA1c* glycated hemoglobin, *HDL* high-density lipoprotein, *LDL* low-density lipoprotein, *Q1* first quartile, *Q3* third quartile

### Weight Loss Outcomes

**Fig. 1:**
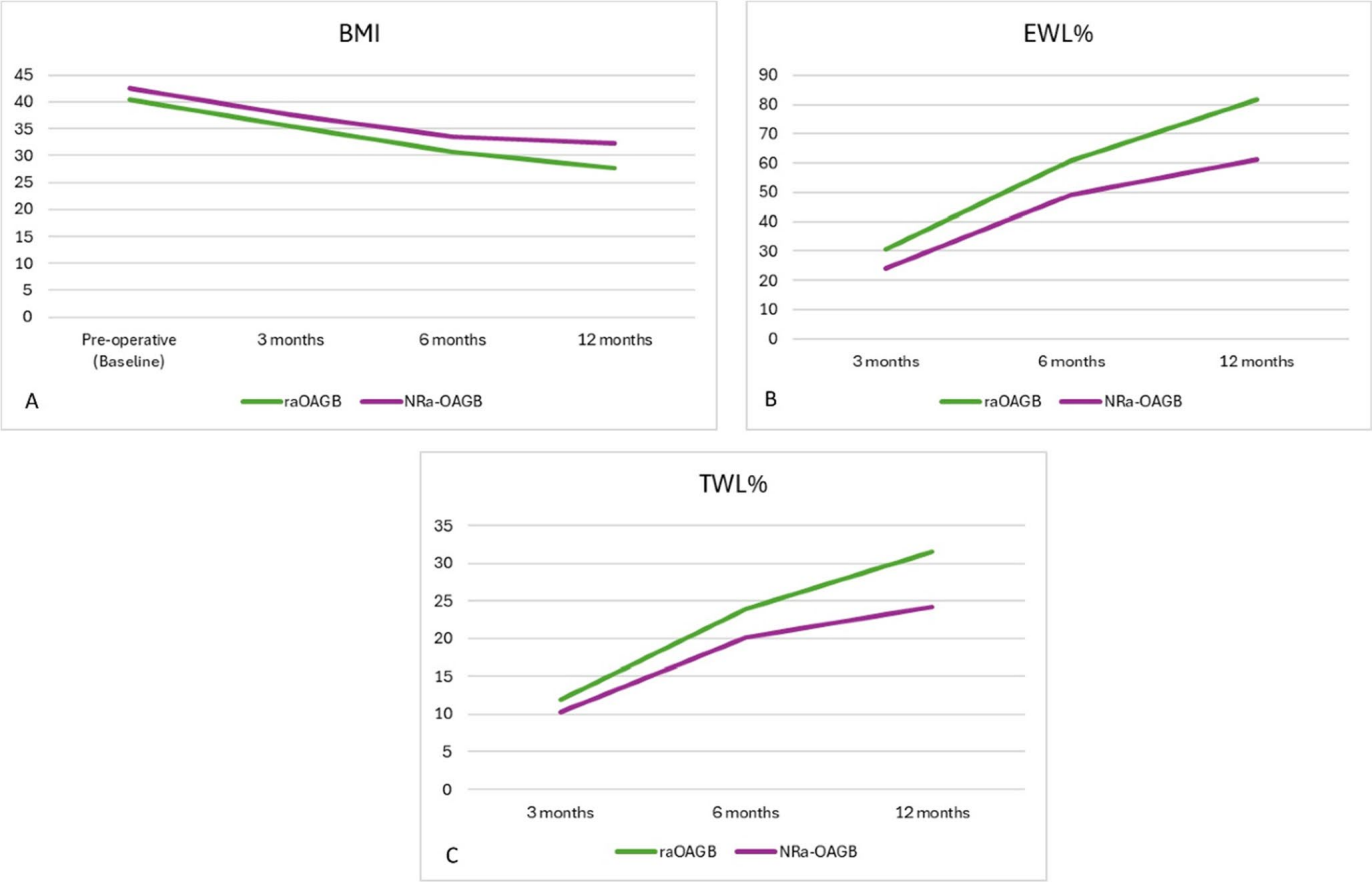
Line graph showing (**A**) median BMI reduction from baseline to 12 months post-revision, (**B**) progressive improvement in %EWL over 12 months, and (**C**) displaying %TWL at 3, 6, and 12 months, showing greater weight-loss efficacy in raOAGB

**Fig. 2:**
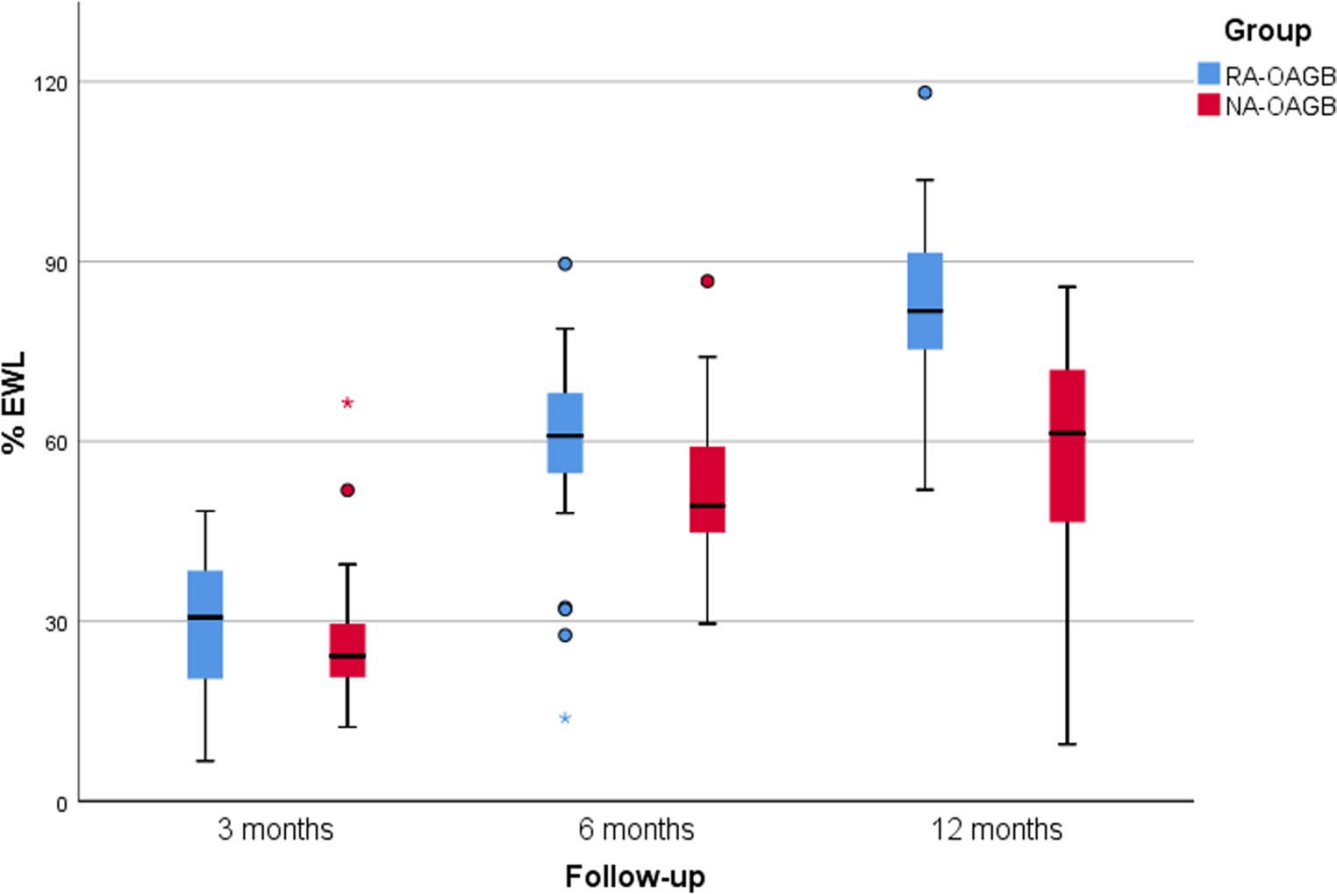
Box-and-whisker plots showing %EWL at 3, 6, and 12 months after revisional surgery, demonstrating consistently greater weight loss in raOAGB compared with NRa-OAGB

Both surgical groups demonstrated significant reductions in BMI from baseline to 12 months (P2 < 0.001) (Table 2; Fig. 1A). raOAGB showed consistently greater reductions than NRa-OAGB, with median BMI at 12 months of 27.6 kg/m² vs. 32.2 kg/m² (P1 < 0.001) (Table 3). Excess weight loss percentage (EWL%) also improved significantly within both groups over time (P2 < 0.001). At 12 months, the raOAGB group achieved a median EWL% of 81.7%, which was significantly higher than the 61.3% reached by the NRa-OAGB group (P1 < 0.001) (Fig. 1B) (Fig. 2). Likewise, total weight loss percentage (TWL%) increased significantly from baseline to 12 months in both groups (P2 < 0.001), but was more pronounced in raOAGB patients, who reached a median TWL% of 31.5%, compared to 24.2% in the NRa-OAGB group (P1 < 0.001) (Table 3) (Fig. 1C).

**Table 3:** Comparison of body mass index (BMI), percentage excess weight loss (EWL%), and percentage total weight loss (TWL%) between ring-augmented OAGB (raOAGB) and non-ring-augmented OAGB (NRa-OAGB) from baseline to 12 months following revisional surgery after sleeve gastrectomy

		Pre-operative (Baseline)	3 months	6 months	12 months	P2-value
BMI	raOAGB	40.5 (38.2–43.6)	35.4 (34.4–38.5)	30.7 (29.7–33.2)	27.6 (26.3–29.8)	**< 0.001**
	NRa-OAGB	42.5 (39.7–45.9)	37.6 (35.4–41.3)	33.5 (30.8–36.5)	32.2 (30.3–34.7)	**< 0.001**
	P1-value	0.066	**0.020**	**0.002**	**< 0.001**	-
EWL%	raOAGB	-	30.6 (19.6–39)	60.9 (54.3–68.2)	81.7 (74.9–91.5)	**< 0.001**
	NRa-OAGB	-	24.2 (20.3–29.8)	49.2 (44.2–59.8)	61.3 (44.8–73.2)	**< 0.001**
	P1-value	-	0.122	**< 0.001**	**< 0.001**	-
TWL%	raOAGB	-	12 (5.6–16.2)	24 (18.5–27.8)	31.5 (27.4–35.6)	**< 0.001**
	NRa-OAGB	-	10.3 (8.9–11.4)	20.2 (18.1–23.2)	24.2 (17.9–30.9)	**< 0.001**
	P1-value	-	0.276	**0.014**	**< 0.001**	-

Values are presented as median (Q1–Q3). P1 denotes between-group comparisons (raOAGB vs. NRa-OAGB) at each time point using the Mann–Whitney U test. P2 denotes within-group comparisons over time (baseline vs. 12 months) using the Wilcoxon signed-rank test

*BMI* body mass index, *EWL%* excess weight loss percentage, *TWL%* total weight loss percentage, *Q1* first quartile,* Q3* third quartile

### Laboratory Outcomes

Baseline hemoglobin levels were slightly higher in the NRa-OAGB group compared to raOAGB (13.2 vs. 12.7 g/dL). At 6 and 12 months, both groups showed mild declines (Table 2). HbA1c levels decreased slightly in both groups. In raOAGB, values declined from 5.4% preoperatively to 5.1% at 12 months, while NRa-OAGB increased from 5.0% to 5.2% (Table 2).

Triglycerides were lower in raOAGB patients at baseline (151 vs. 195 mg/dL) and remained lower at 12 months (136 vs. 150 mg/dL) (Table 2). Total cholesterol was higher in NRa-OAGB at baseline (222 vs. 186 mg/dL), but the difference disappeared at 12 months (167 vs. 166 mg/dL) (Table 2). HDL cholesterol increased in both groups; raOAGB showed a gradual rise from 53.1 to 62 mg/dL, while NRa-OAGB patients experienced a more pronounced increase, reaching 101 mg/dL at 12 months (Table 2). The increase in the NRa-OAGB group should be interpreted cautiously due to lower and more variable baseline HDL and the use of medians from a small retrospective sample. LDL cholesterol decreased similarly in both groups, remaining at 89 mg/dL after 12 months. Vitamin B12 levels were similar between groups at baseline (310 vs. 320 pg/mL) (Table 2). Both groups demonstrated stable levels. Calcium levels were slightly higher in NRA-OAGB preoperatively (9.2 vs. 8.9 mg/dL) (Table 2).

**Fig. 3:**
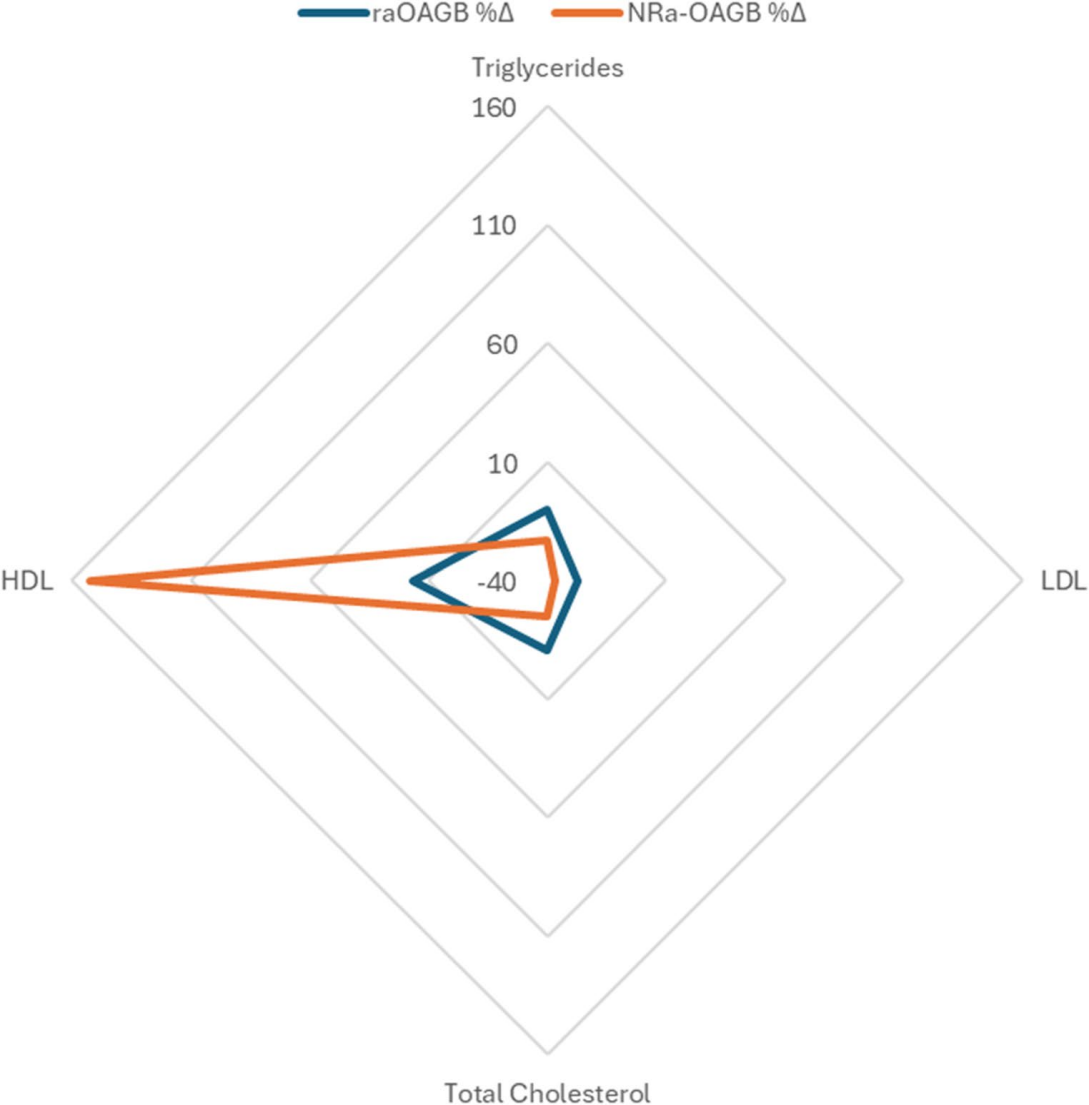
Radar chart showing relative percentage changes in lipid parameters at 12 months compared with baseline, where both groups demonstrated metabolic improvements

The radar chart illustrates the median percentage change in lipid profile parameters from baseline to 12 months following raOAGB and NRa-OAGB. raOAGB demonstrated moderate improvements across triglycerides, LDL cholesterol, and total cholesterol, while NRa-OAGB showed a disproportionately high relative increase in HDL levels due to markedly lower baseline values. Axes scaled from − 40% to + 160% to ensure visibility across parameters. Data expressed as median % change (Fig. 3).

### Improvement in Obesity related conditions

At 12 months, improvements in type 2 diabetes, hypertension, obstructive sleep apnea, and dyslipidemia were observed in both groups, but none of the inter-group comparisons reached statistical significance (all *p* > 0.05) (Table 4).

**Table 4:** Improvement in obesity-associated comorbidities at 12 months following revisional ring-augmented OAGB (raOAGB) and non-ring-augmented OAGB (NRa-OAGB)

		raOAGB	NRa-OAGB	Test statistic	*P*-value
		(*n* = 45)	(*n* = 49)		
Type 2 Diabetes Mellitus		10 (22.2)	5 (10.2)	FE(𝒳^2^ = 2.526)	0.159
Hypertension		15 (33.3)	14 (28.6)	FE(𝒳^2^ = 0.249)	0.660
Obstructive sleep apnea		4 (8.9)	3 (6.1)	FE(𝒳^2^ = 0.260)	0.706
Dyslipidemia		15 (33.3)	13 (26.5)	FE(𝒳^2^ = 0.519)	0.505

Values are presented as n (%), representing patients who demonstrated remission or clinically relevant improvement of the respective comorbidity at 12 months compared with baseline, according to treating physician assessment and standard clinical criteria. P-values refer to between-group comparisons using Fisher’s exact test

*T2DM* type 2 diabetes mellitus

### Operative and Postoperative Outcomes

Median operative time was similar between groups (111 min in raOAGB vs. 113 min in NRa-OAGB, *p* = 0.636). Length of hospital stay was identical (median 3 days, *p* = 0.610) (Table 5). Early postoperative complications were rare: one case of bleeding occurred in NRa-OAGB (2%) and none in raOAGB (*p* = 1.000) (Table 5). Food intolerance was reported in 1 (2.2%) of raOAGB and 0 (0%) of NRa-OAGB patients (*p* = 0.479) (Table 5). Late nutritional deficiencies occurred in 4 (8.9%) of raOAGB and 1 (2%) of NRa-OAGB (*p* = 0.190), without statistical significance (Table 5). Ring removal was reported in 1 (2.2%) of raOAGB and none of the NRa-OAGB patients (*p* = 0.479) (Table 5). In the single raOAGB patient requiring ring removal, the indication was persistent vomiting and food intolerance, and removal was performed 10 months postoperatively.

**Table 5:** Intraoperative characteristics and postoperative outcomes of ring-augmented OAGB (raOAGB) versus non-ring-augmented OAGB (NRa-OAGB), including operative time, hospital stay, early complications, late nutritional deficiencies, and ring-related events

	raOAGB	NRa-OAGB	Test statistic	*P*-value	
	(*n* = 45)	(*n* = 49)			
Operative time ^*^	111 (92.5–121)	113 (91–123)	U = 1165	0.636	
Hospital stays ^*^	3 (2–3)	3 (2–3)	U = 1167	0.610	
Early complications					
Bleeding	0 (0)	1 (2)	FE(𝒳^2^ = 0.928)	1.000	
Food intolerance	1 (2.2)	0 (0)	FE(𝒳^2^ = 1.485)	0.479	
Ring removal	1 (2.2)	0 (0)	FE(𝒳^2^ = 1.485)	0.479	
Late complications					
nutritional deficiency	4 (8.9)	1 (2)	FE(𝒳^2^ = 2.184)	0.190	

^a^ median (Q1 - Q3). Continuous variables are presented as median (Q1–Q3) and were compared between groups using the Mann–Whitney U test. Categorical variables are presented as n (%) and were compared using Fisher’s exact test. Early complications are defined as events occurring within 30 days after surgery; late complications occur beyond 30 days. Nutritional deficiency refers to clinically significant micronutrient or hematologic abnormalities requiring therapeutic intervention

*Q1* first quartile, *Q3* third quartile

## Discussion

This study compared the outcomes of raOAGB versus NRa-OAGB as revisional procedures following RWG after primary SG. Both techniques achieved significant weight loss and metabolic improvements at 12 months, but important differences emerged that highlight the potential advantages of RA.

### Weight Loss Efficacy

raOAGB demonstrated significantly greater reductions in BMI, EWL%, and TWL% compared to NRa-OAGB at 12 months. The NRa-OAGB cohort had a higher baseline disease burden, with more hypertension and GERD, which could influence recovery, adherence, and follow-up, and thereby affect weight-loss trajectories. Given the retrospective, non-randomized design, these imbalances may introduce residual confounding; therefore, the between-group differences should be interpreted as associative rather than definitively causal. The gastric ring acts as a restrictive element and may contribute to sustained weight loss by supporting outlet control and limiting functional enlargement over time; however, this mechanistic interpretation remains speculative because pouch morphology was not directly assessed in our cohort. Longer-term evidence from other MBS contexts has reported improved weight maintenance and reduced RWG compared with non-augmented counterparts [17, 28–31]. At the same time, long-term outcomes are not uniform across trials, and ring-related intolerance, including dysphagia and occasional ring removals, has been reported; therefore, extrapolation to revisional raOAGB should be made cautiously [13]. OAGB-specific long-term comparative evidence is still emerging and is being addressed by ongoing trials evaluating banded OAGB.

### Metabolic and Laboratory Outcomes

Both groups exhibited favorable lipid changes, but with distinct profiles. Importantly, LDL cholesterol declined in both cohorts, and total cholesterol equalized by 12 months, underscoring the metabolic efficacy of OAGB irrespective of augmentation. Glycemic control, reflected by HbA1c, did not differ significantly, likely due to relatively low baseline values and the short follow-up duration. It is important to acknowledge that the degree of weight loss itself is the principal determinant of improvement in the metabolic profile after MBS, irrespective of the surgical technique employed. Greater reductions in body mass index and adiposity lead to more pronounced enhancements in insulin sensitivity, hepatic glucose regulation, and lipid metabolism [32]. As visceral fat decreases, free fatty acid flux into the liver is reduced, thereby lowering triglyceride synthesis and improving lipoprotein balance [32]. At the same time, diminished adipose-driven inflammation and improved peripheral insulin action contribute to reductions in HbA1c [33].

Specific surgical procedures may differ in their anatomical alterations and hormonal effects, but the magnitude of weight loss achieved remains the strongest predictor of durable improvements in glycemic control and triglyceride levels [32, 34]. While the current study’s follow-up is limited to 12 months, longer-term studies on ring-augmented procedures have indicated sustained metabolic benefits, suggesting that the enhanced weight maintenance observed with ring augmentation could translate into more durable metabolic improvements [28].

### Perioperative Outcomes and Complications

Improvements in hypertension, diabetes, sleep apnea, and dyslipidemia were observed in both groups. The lack of significant inter-group differences may reflect sample size limitations, as baseline conditions burdens were unevenly distributed. Nonetheless, the enhanced weight loss in raOAGB could translate into superior long-term obesity-related conditions with extended follow-up [29].

Median operative time and length of hospital stay were similar between groups, indicating that the addition of a gastric ring does not significantly increase the immediate surgical time. Early postoperative complications were rare and comparable between groups. Although ring-related complications are recognized in augmented procedures, only one patient (2.2%) in the raOAGB group required ring removal due to persistent vomiting and food intolerance 10 months postoperatively. No cases of ring migration or erosion were observed during the study period.

While this study reported low rates of food intolerance and late nutritional deficiencies without statistical significance, other studies on ring-augmented procedures have noted potential complications such as ring erosion, slippage, and dysphagia, although often at low rates [28, 35]. For instance, one study on raRYGB reported ring erosion rates ranging from 0% to 7.7% and other ring-related complications like slippage and herniation [35]. The careful, loose positioning of the MiniMizer Gastric Ring^®^ in the raOAGB group aims to minimize these risks by maintaining mechanical restriction without compromising gastric compliance [36]. This approach is crucial for balancing the benefits of enhanced restriction with the potential for adverse events.

### Food Tolerance and Dumping Syndrome

The current study’s findings on food intolerance did not show a significant difference between the groups at 12 months. However, research on raRYGB has shown interesting temporal patterns in food tolerance and dumping syndrome. Initially, no significant differences were observed, but at longer follow-up, the non-ring-augmented group demonstrated a significantly higher incidence of dumping syndrome [29]. This suggests that ring augmentation may promote more consistent food passage and regulate gastric emptying over time, potentially reducing dumping frequency despite potentially causing greater food intolerance in some patients. This complex relationship highlights the need for longer-term follow-up to fully understand the impact of ring augmentation on patient quality of life and gastrointestinal symptoms.

### Revisional Context

This study specifically addresses revisional OAGB after SG. The initial SG procedure often leads to anatomical and physiological changes, such as sleeve dilation or neo-fundus development, contributing to SOWL or RWG [37, 38]. OAGB has emerged as a preferred revisional option due to its efficacy and metabolic benefits [39, 40]. The addition of ring augmentation in this revisional context is conceptually intended to enhance weight maintenance through outlet control; however, whether ring augmentation specifically counteracts the anatomical drivers of SG failure cannot be established from our dataset, as sleeve morphology and pouch/outlet dimensions were not systematically assessed [29]. The positive outcomes observed in raOAGB in this study provide preliminary evidence supporting its role in improving revisional surgery results, aligning with the theoretical benefits of mechanical restriction in supporting longer-term weight maintenance rather than definitively preventing anatomical change [30, 41].

Notably, the specific mechanisms of SOWL and RWG after index SG were not systematically captured in this retrospective dataset, and many procedures were performed outside our center, leading to inconsistent anatomical and technical data for reliably determining the cause of failure. Thus, our findings do not imply that ring augmentation counters a specific mechanism but rather show comparative 12-month outcomes in a real-world revisional cohort.

### Strengths and Limitations

A key strength of this study is that it addresses a novel and underexplored topic: the role of ring augmentation in revisional one-anastomosis gastric bypass following primary MBS. To our knowledge, few studies have systematically compared ring-augmented versus non-augmented OAGB in this specific revisional context. By directly evaluating weight loss, metabolic outcomes, obesity-related conditions improvement, and safety, this work provides early evidence to guide surgical decision-making in an area of growing clinical relevance.

However, several limitations should be acknowledged. Firstly, its retrospective design inherently introduces potential biases, including selection bias and information bias, as data were collected from existing records rather than through a controlled, prospective protocol. While efforts were made to ensure data quality from a prospectively maintained institutional database, the absence of randomization means that unmeasured confounders could influence the observed outcomes. For instance, the NRa-OAGB group had a significantly higher proportion of ASA III patients and a greater prevalence of hypertension and GERD at baseline, indicating a potentially sicker cohort, which could impact postoperative outcomes independently of the surgical technique. In addition, hiatal hernia was clinically assessed and repaired when found, but its presence was not standardized as a study variable, limiting group-level frequency reporting. Objective assessments of sleeve volume and shape were not conducted, as the study focused on comparing the safety and efficacy of revisional raOAGB versus non-raOAGB rather than anatomical characteristics or the causes of RWG after SG. Additionally, it was challenging to classify the reasons for revision since many index SG procedures were performed outside our institution, leading to inconsistent documentation. Therefore, we couldn’t determine if ring augmentation benefits specific failure phenotypes.

Secondly, the relatively short follow-up period of 12 months limits the ability to draw definitive conclusions regarding the long-term durability of weight loss and metabolic improvements, as well as the incidence of late complications. MBS outcomes, particularly RWG and the emergence of certain complications like ring erosion or nutritional deficiencies, can manifest years post-procedure [35]. Therefore, the observed advantages of raOAGB in terms of weight loss efficacy at 12 months may not necessarily translate into sustained long-term superiority, and the full spectrum of ring-related complications might not yet be apparent.

Thirdly, the study was conducted at a single high-volume tertiary MBS center by a single experienced surgical team. While this ensures consistency in surgical technique and postoperative care, it may limit the generalizability of the findings to other institutions with different patient populations, surgical volumes, or levels of expertise. The specific patient selection criteria and institutional protocols could also influence outcomes, making direct comparisons with studies from diverse settings challenging.

Finally, the sample size might limit the statistical power to detect subtle but clinically significant differences, especially for less common complications or specific metabolic parameters. Larger, multicenter, randomized controlled trials with extended follow-up periods are needed to confirm these findings and provide more robust evidence regarding the optimal revisional strategy after primary MBS.

### Future Directions

Building upon the findings of this study and addressing its limitations, several avenues for future research emerge to further elucidate the role of ring augmentation in revisional bariatric surgery.

Firstly, long-term prospective randomized controlled trials are critically needed to compare raOAGB and NRa-OAGB with extended follow-up periods. Such trials would provide robust evidence on the durability of weight loss, sustained metabolic improvements, and the true incidence and management of late complications, including ring-related issues like erosion, migration, and the need for re-intervention. These studies should also meticulously track nutritional deficiencies and quality of life metrics to provide a holistic view of patient outcomes.

Secondly, mechanistic studies are warranted to explore the precise physiological mechanisms by which ring augmentation contributes to enhanced weight loss and maintenance. This could involve advanced imaging techniques to monitor gastric pouch and gastrojejunostomy dimensions over time, as well as hormonal assays to understand the impact of the ring on satiety-regulating hormones (e.g., ghrelin, GLP-1, PYY). Understanding these mechanisms could lead to further refinements in surgical technique and patient selection. In parallel, ongoing randomized trials in ring-augmented OAGB will be critical to clarify long-term durability and ring-related adverse events in the OAGB setting [23].

Thirdly, research should focus on identifying optimal patient selection criteria for raOAGB. Subgroup analyses could investigate whether certain patient characteristics (e.g., initial BMI, presence of specific obesity-related conditions, psychological factors, or eating behaviors) predict a greater benefit from ring augmentation. This would allow for a more personalized approach to revisional MBS.

Finally, cost-effectiveness analyses comparing raOAGB and NRa-OAGB are essential. While ring augmentation may offer superior long-term weight control, the initial cost of the device and potential for ring-related complications need to be weighed against the long-term health benefits and reduced healthcare expenditures associated with sustained weight loss and resolution of obesity-related diseases. This would provide valuable information for healthcare policymakers and patients alike.

## Conclusion

This comparative cohort study suggests that raOAGB is a safe and effective revisional option after SG-associated SOWL or RWG, achieving superior 12-month weight loss versus non-augmented OAGB without prolonging operative time. Ring-related adverse events were uncommon, including one ring removal. Longer-term prospective studies are needed to confirm durability and late ring-related outcomes before routine recommendation.

## Supplementary Information

Below is the link to the electronic supplementary material.

Supplementary Material 1 (MP4 59.2 MB)

## Data Availability

The datasets generated and analyzed during the current study are available from the corresponding author upon reasonable request.

## Abbreviations

ASA: American Society of Anesthesiologists
BMI: Body Mass Index
EWL%: Excess Weight Loss Percentage
GERD: Gastroesophageal Reflux Disease
HbA1c: Glycated Hemoglobin
HDL: High–Density Lipoprotein
IQR: Interquartile Range
LDL: Low–Density Lipoprotein
MBS: Metabolic and Bariatric Surgery
NRa: OAGB–Non–Ring–Augmented One–Anastomosis Gastric Bypass
OAGB: One–Anastomosis Gastric Bypass
PDS: Polydioxanone Suture
PPI: Proton Pump Inhibitor
RA: Ring Augmentation
RaOAGB: Ring–Augmented One–Anastomosis Gastric Bypass
RWG: Recurrent Weight Gain
SD: Standard Deviation
SG: Sleeve Gastrectomy
SOWL: Suboptimal Weight Loss
STROBE: Strengthening the Reporting of Observational Studies in Epidemiology
T2DM: Type 2 Diabetes Mellitus
TWL%: Total Weight Loss Percentage
