# Publisher Correction: Ring-Augmented versus Non-Ring-Augmented One-Anastomosis Gastric Bypass as Revisional Surgery after Sleeve Gastrectomy: a Comparative Cohort Study

**DOI:** 10.1007/s11695-026-08700-7

**Published:** 2026-04-25

**Authors:** Ahmed Elmasry, Mohamed Sallam, Mahmoud F. Abdelaziz, Amr Abdelatyy, Mohamed Abdelgawad, Muhammad S. Sahali, Eslam E. Mohamed, Hashem Altabbaa, Ahmed El Shamarka, Mohamed H. Zidan

**Affiliations:** 1 Arab Contractors Medical Centre, Cairo, Egypt; 2 Independent Surgeon, Ministry of Health and Population, Cairo, Egypt; 3 Matareya Teaching Hospital, Cairo, Egypt; 4 Al-Basheer-Hospital, Amman, Jordan; 5 The Research Papyrus Lab, Alexandria, Egypt; 6 Alexandria University, Alexandria, Egypt

**Publisher Correction: Obesity Surgery**

10.1007/s11695-026-08616-2

The original article has been updated to add a missing author name.

